# Dietary Intake Associated with Periodontitis in Adults: A Scoping Review

**DOI:** 10.3390/nu18213537

**Published:** 2026-10-27

**Authors:** Nydia Y. Sanchez-Orozco, Miguel A. Robledo-Valdez, Alondra Ruiz-Gutierrez, Carmen de la Rocha, Fabiola Márquez-Sandoval, Lucrecia Carrera-Quintanar

**Affiliations:** 1 Universidad de Guadalajara, Centro Universitario de Ciencias de la Salud, Departamento de Alimentación y Nutrición, Doctorado en Ciencias de la Nutrición Traslacional, Guadalajara 44340, Jalisco, Mexico; nydiasanchez.oro@gmail.com (N.Y.S.-O.); miguel.robledo3711@alumnos.udg.mx (M.A.R.-V.); carmen.martindc@academicos.udg.mx (C.d.l.R.); 2 Universidad de Guadalajara, Centro Universitario de Ciencias de la Salud, Departamento de Clínicas Odontológicas Integrales, Especialidad en Periodoncia, Guadalajara 44340, Jalisco, Mexico; alondra.rgutierrez@academicos.udg.mx; 3 Universidad de Guadalajara, Centro Universitario de Ciencias de la Salud, Departamento de Clínicas de la Reproducción Humana, Crecimiento y Desarrollo Infantil, Instituto de Investigación en Cáncer en la Infancia y Adolescencia, Guadalajara 44340, Jalisco, Mexico

**Keywords:** periodontitis, nutrients, diet components, eating patterns

## Abstract

**Background:** Periodontitis continues to be a global public health burden that is associated with an increased risk of metabolic diseases such as type 2 diabetes mellitus, metabolic syndrome, and cardiovascular diseases. Several studies, unsystematized, have evaluated the relationship between consumption of specific foods, nutrients, or eating patterns and the disease, aiming to support personalized and holistic periodontal therapy. **Objective:** This scoping review aimed to evaluate which dietary intakes are linked to the pathology. **Methods:** The scoping review followed the Preferred Reporting Items for Systematic Reviews and Meta-Analyses extension for Scoping Reviews (PRISMA-ScR) guidelines. A systematic search was conducted in PubMed. The inclusion criteria were original studies with adult populations that evaluated diet and nutrient intake in populations with periodontitis. **Results:** Thirty-six articles were included (26 cross-sectional, 6 cohort studies, 3 randomized clinical trials, and 1 case–control). Specifically, adequate protein intake significantly reduced bleeding on probing, while high fiber intake was inversely associated with alveolar bone loss and tooth loss. Furthermore, higher consumption of vitamins C and D correlated with a reduced periodontitis risk. Negative associations were observed between nutrient groups (vitamin A, C, E, K, flavonoid, fiber) and plant-based food groups (green–yellow vegetables, general vegetables, fruit) and community periodontal index, tooth loss, probing depth, and clinical attachment loss. Broader diet quality also mattered: adherence to healthy anti-inflammatory dietary patterns were consistently associated with lower periodontitis risk, whereas pro-inflammatory patterns were in the opposite direction. **Conclusions:** This review strengthens the importance of the links between single nutrients, food groups, and eating patterns in periodontitis. Integrating personalized nutritional advice into dental settings represents a valuable host-modulating strategy to optimize periodontal therapy outcomes.

## 1. Introduction

Periodontal tissues, including the root cementum, periodontal ligament, alveolar bone, and gingiva, support and protect dental organs, nerves, and blood vessels from injury [1]. In accordance with the American Dental Association (ADA) guidelines and the 2017 World Workshop Classification of Periodontal and Peri-Implant Diseases and Conditions, periodontitis is defined as a progressive chronic inflammatory disease characterized by clinical attachment loss (CAL), probing pocket depth (PPD), and radiographically assessed alveolar bone destruction, which can ultimately lead to tooth loss (TL) [2]. Subgingival dysbiotic biofilm interacts with a susceptible host immune response to drive disease pathogenesis, a process modified by local, genetic, environmental, and behavioral factors [3,4]. Globally, severe periodontitis affects approximately 11% of the adult population, representing over 700 million people, making it a major public health burden [3,5,6].

Severe periodontitis is associated with several systemic diseases because it is a multifactorial chronic inflammatory disease that triggers a series of host immunological and inflammatory responses [7]. These processes can lead to adverse pregnancy outcomes, in addition to increasing the risk of respiratory diseases (chronic obstructive pulmonary disease, asthma, and pneumonia) [8], type 2 diabetes mellitus (DM), metabolic syndrome (MS) [9], cardiovascular diseases (CVD), or endothelial dysfunction [10,11], and Alzheimer/dementia diseases [12,13]. This is because elevated salivary matrix metalloproteinases and proinflammatory cytokines can migrate into the systemic circulation and trigger neuroinflammatory responses [11,14,15].

Nutrition is widely recognized as a cornerstone of preventing and managing non-communicable chronic inflammatory conditions, including periodontitis [16]. Adequate periodontal health depends on oral hygiene and dental care, as well as biological, social, and behavioral factors and the individual’s nutritional status. In this context, diet is a key modifiable factor that can mitigate oxidative stress, systemic inflammation, and subgingival dysbiosis, mainly driven by Gram-negative anaerobic genera (*Porphyromonas gingivalis*, *Treponema denticola*, *Tannerella forsythia*) that characterize periodontitis [17,18,19]. For these reasons, nutrition-based treatment has been suggested as an adjunctive therapy to periodontal management [20].

Despite multiple investigations and publications, unsystematized, no consensus exists on nutritional therapy that can contribute to the prevention and/or treatment of periodontitis, and limited information is available to inform the development of precision nutrition guides.

Therefore, this scoping review aimed to systematically evaluate the association between habitual dietary intake and clinical periodontitis parameters through a three-tiered analytical framework, excluding randomized clinical trials that used supplements or nutraceuticals.

## 2. Materials and Methods

To provide an overview of this topic, we conducted a scoping review [21,22]. The role of nutrition in preventing and/or treating periodontitis remains unclear, underscoring the need to identify knowledge gaps to accelerate further clinical studies in this field [23]. The scoping review followed the Preferred Reporting Items for Systematic Reviews and Meta-Analyses extension for Scoping Reviews (PRISMA-ScR) guidelines [24]. The corresponding checklist used for the development is included (see Supplementary Table S1).

### 2.1. Search Strategy

We performed a systematic search in PubMed to identify original English-language publications, including a human population aged 18 years or older, from January 2012 to June 2026. The search terms used in this study were: Food [MeSH Terms] AND periodontal disease [MeSH Terms]; “diet” OR “nutrients” AND “periodontal disease”.

This scoping review aimed to evaluate which dietary nutrients are linked to periodontitis to answer the question using the PCC framework (population, concept, and context): What dietary intakes are associated with clinical variables of periodontal disease in adults, where P (adults with periodontitis), C (dietary intake), and C (adults without systemic diseases)?

### 2.2. Eligibility Criteria

This review included published studies on dietary and/or food intake in relation to periodontitis. The inclusion criteria were (1) original studies (clinical trials, observational studies) involving adults over 18 years of age, (2) studies which the main purpose were evaluated dietary intake of food and nutrient; i.e., no supplementation, (3) studies published in the English language, (4) studies in healthy populations (without systemic diseases) or with periodontitis, (5) studies with self-report of periodontitis or that evaluated at least one periodontal clinical parameter, for example, alveolar bone loss (ABL), pocket probing depths (PPD), clinical attachment loss (CAL), percentage of sites with bleeding on probing (BOP), gingival index (GI), community periodontal index (CPI), etc., or periodontitis diagnostics, and (6) studies published in a time period from January 2012 to June 2026.

### 2.3. Exclusion Criteria

The present review mainly excluded studies with subjects with gingivitis, meta-analysis, conference abstracts, narrative reviews, book chapters, perspectives and commentaries, articles that did not discuss dietary intake or that used any drug, supplement or mouthwash, experimental studies that were in vitro or in vivo, as well as studies in vulnerable or specific populations with some disease (pregnant women, adolescents, children or populations with obesity, DM, MS, CVD, autoimmune diseases or others).

### 2.4. Selection Process

Duplicate articles were excluded. We independently screened titles and/or abstracts for potentially eligible studies and excluded irrelevant studies. Subsequently, the full texts of studies shortlisted for inclusion were independently reviewed, and the reasons for excluding any studies are shown. Likewise, articles that met the inclusion criteria but did not appear in the search terms were added as independent searches.

### 2.5. Data Extraction

Data were extracted independently from the included studies using a standardized data extraction form. Two reviewers conducted the data extraction process to reduce the risk of bias. The form was developed in a Microsoft Excel spreadsheet to extract the relevant information from each study. The extracted information included the study characteristics (authors, year, journal, impact factor, study design, title, study objective, population characteristics, periodontal disease, measurement instruments, nutrient evaluated, main results, and conclusions).

The results section below describes the most relevant scientific findings on nutritional components and their roles in the development, prevention, and treatment of periodontitis.

## 3. Results

### 3.1. Study Results

This search for studies yielded articles from both searches in the PubMed electronic database. We found and removed 34 duplicates. After removing duplicates, we removed another 966 articles during title and abstract screening. We then reviewed the full texts of 83 shortlisted articles for inclusion and excluded 54 for not meeting the inclusion criteria. Subsequently, 29 articles that met the eligibility criteria were included. In addition to the database search, the authors identified seven relevant articles through their prior domain expertise and familiarity with the field. The initial PubMed query missed these studies because of variations in key terminology or indexing delays. To maintain methodological rigor and prevent selection bias, we formally screened all seven studies against the same eligibility criteria applied to the primary database search. A total of 36 articles were included in this review. Figure 1 summarizes the study identification process.

### 3.2. General Description of Results

Of the publications found under the search criteria, 36 meet the inclusion criteria. Overall, the more representative year of publication was 2021 with 10 reports included, perhaps due to the delay associated with the COVID-19 pandemic [25], followed by 2022 and 2023 with four publications identified this year, 2018, 2020 and 2025 with three per year, two per year in 2016 and 2017, one per year in 2012–2015 and 2024, and none in 2019 and 2026 met the criteria, indicating a trend in increasing interest in the research area which suggests a growing concern about understanding and addressing this health issue. Most selected studies used a cross-sectional design (n = 26, 72%), followed by cohort studies (n = 6, 17%) and randomized clinical trials (n = 3, 8%); case–control studies were least represented (n = 1, 3%) (Figure 2).

Of the selected articles, the most represented populations were North Americans (18 studies) and Europeans (12 studies), followed by Asians (six studies) and finally, South Americans (one study); it needs to be noted that one study included both American and British populations [26]. No other populations were studied in the selected publications. Most of the investigations included healthy populations (n = 28; 77%), and less than 25% included patients with periodontitis (22%; n = 8). The results are presented by dietary parameters and organized as follows: single nutritional component (Table 1), group of nutrients or foods (Table 2), and eating patterns (Table 3), and are described in the following paragraphs.

#### 3.2.1. Single Nutritional Components and Periodontitis

When segregating studies that evaluate a single nutrient (Table 1), we identified eight publications, of which five were cross-sectional, one cohort, and two randomized clinical trials; both the cross-sectional and cohort designs included healthy volunteers and were then classified according to the oral measurements of periodontitis or not, while the clinical trials included patients with periodontitis; the populations included were from America (n = 4 Americans; n = 1 Canadian), Asia (n = 1 Korean), and Europe (n = 1 German; n = 1 Italian).

Cross-sectional studies have addressed the relationship between periodontal disease and protein intake [30], vitamin C [29], vitamin D [27], fiber [28], and whole and refined grains [31]. One cohort study also evaluated fiber’s association with periodontitis over time [32]. In all cases, the adequate intake of these nutrients and fiber (each evaluated separately) was associated with lower risk or lower periodontal indicator scores.

Of the selected clinical trials, one examined the effect of short-term vitamin C from kiwi [33], while the other assessed the impact of sugar restriction [34]. In Graziani et al. [33], patients with periodontitis were given kiwifruit twice daily for two months. After that, patients received periodontal treatment (scaling and root planing) and continued the dietary intervention for another 3 months, with adherence evaluated by self-report. The intervention group showed a positive effect by significantly reducing the BOP and PI, but this benefit was observed only before periodontal intervention. In contrast, Pappe et al. [34]., in their clinical trial, observed changes in the clinical parameters BOP, PCR, and GBI in both study groups (avoidance of added sugar consumption and those with their usual intake); no differences were observed between the study groups.

#### 3.2.2. Group of Nutrients or Foods and Periodontitis

Regarding the relationship between groups of nutrients or multiple components of the diet and periodontal parameters, 10 studies were included (Table 2): six cross-sectional, one randomized clinical trial, two cohort studies and one case–control study. These studies include populations from North America (n = 2 Americans; n = 2 Canadians), Europe (n = 1 German; n = 1 British), and Asia (n = 2 Japanese; n = 2 Koreans). For this group, most studies include ill subjects (six studies include patients with periodontitis), and the rest (cross-sectional designs) include healthy volunteers.

Among the cross-sectional studies, the results focused on the relationship between periodontitis and macro- and micronutrient intake [35]; general dietary habits [36]; plant-based foods [37]; the joint intake of phosphorus, riboflavin, thiamine, niacin, vitamin C, calcium, protein, and carbohydrates [38]; vitamin K and fiber intake [39]; and Vitamins A, B1, B2, B6, B12, C, D, E, and K [40]. More consistent findings were a low risk of periodontitis with the intake of vitamin E and A, green and yellow vegetables, fruits, phosphorus, and carbohydrates [36,37,38,40]. Furthermore, vitamin K and fiber intake were negatively associated with CAL progression. Hamasaki et al. [35] and Hwang et al. [38] observed, through a multivariate analysis, a positive association between fat consumption and disease severity.

In the identified case–control report, the periodontal group had higher daily intakes of vitamin C, DPA, and omega-3 fatty acids [41]. To corroborate this result, serum concentrations were measured, revealing higher levels of DPA and vitamin C in patients with periodontitis. In cohort studies, one examined the relationship between periodontitis and the consumption of fruits, vegetables, and nutrients with antioxidant and anti-inflammatory capacity, with 1-year [42] and 4-year [43] follow-ups. Dodington et al. [42] found a negative association between the intake of β-carotene, vitamin C, and EPA + DHA and periodontitis, and Sparrow et al. [43] found a negative association between the flavonoid intake and PPD.

As mentioned earlier, we included one randomized clinical trial that examined the effects of increasing fruit and vegetable intake on clinical parameters in periodontitis. A six-month intervention that increased fruit, vegetable, and whole-grain intake did not affect the periodontal clinical parameters in patients with periodontitis compared with the control group [44].

#### 3.2.3. Eating Patterns and Periodontitis

Regarding the dietary patterns and periodontitis, we selected 18 articles (Table 3), comprising 15 cross-sectional and 3 cohort studies. Among the 13 cross-sectional studies evaluating the association with periodontitis, the assessed patterns included a posteriori dietary patterns reflecting micronutrient intake [49,50,55,59], pro-inflammatory diets [51,52,56,57], the Nordic diet [45,46], the Dietary Approach to Stop Hypertension (DASH) [53], the Alternative Healthy Eating Index [47], and the Healthy Eating Index [26]. Geographically, these studies represent populations from North America (n = 9; Americans), Europe (n = 8; Germans: n = 2, British: n = 2 (one mixed with Americans), Norway (n = 1, Finns: n = 3), Asia (n = 1; Koreans), and South America (n = 1; Brazilians). Most investigations were conducted in systemically healthy subjects [40,45,46,47,48,49,50,52,55,56,57,58,60,61]. Notably, while most studies excluded or adjusted for smoking, Jauhiainen et al. (2018) specifically evaluated daily smokers [46]. One study also focused on the periodontitis population, with the diagnosis based on a quantitative indicator [54]. The primary findings are described below.

The most relevant results from these cross-sectional studies were as follows: regarding diet quality, a higher Alternative Healthy Eating Index (AHEI), better diet quality, was associated with a lower risk of severe periodontitis [47]. Higher intakes of energy, zinc, fiber, iron, magnesium, manganese, phosphorus, potassium, and omega-3 were also associated with a healthy periodontal status [50]. Across dietary patterns, the results showed that patterns high in micronutrients and fiber [55] and high in fruits and vegetables [59] were inversely associated with the risk of periodontitis. In contrast, patterns high in fat increased the disease risk [55]. The high-refined cereals and desserts pattern was also associated with a higher risk of having fewer teeth [59]. In turn, a dietary pattern rich in salads, fruits, vegetables, poultry, seafood, water, or drinking tea was associated with a lower CAL [49]. One study examining a plant-based diet reported an inverse association between the hPDI z-scores (healthy plant-based diet index) and periodontitis [57].

The DASH and Mediterranean diet patterns were associated with a low risk of periodontitis [53]. For its part, the Nordic diet with the higher RFDS (Recommended Finnish Diet Score) was associated with less gingival bleeding in young people with poor oral hygiene and in adults with poor hygiene; both RFDS and BSDS (Baltic Sea Diet Score) were inversely associated with less gingival bleeding and with the number of teeth with PPD [45]. In smokers, no association was found with any pattern [46]. The AHEI and A Priori Diet Quality Score (APDQS) were not associated with periodontitis; however, high adherence to APDQS resulted in an inverse relation for %BOP [54]. Regarding pro-inflammatory diets, high Energy-adjusted Dietary Inflammatory Index (E-DII) consumption was related with a higher risk of periodontitis [51,56]. Machado et al. and Lieske et al. also reported a positive association between anti-inflammatory diet scores and periodontal clinical parameters [52,58], strengthening the association between inflammatory diets and periodontitis.

Across three cohort designs examining the association between diet quality and oral health, poor diet quality was associated with fair/poor oral health and periodontitis, and a low Elderly Dietary Index (EDI) was associated with tooth loss [26]. Furthermore, Prudent and Western dietary patterns and empirical dietary inflammatory patterns (EDIP) were not associated with periodontal disease risk. Nevertheless, the risk of periodontitis was higher in the group of obese individuals in the Western pattern [60,61].

## 4. Discussion

This scoping review collects and analyzes evidence on food intake and periodontitis indicators, excluding randomized clinical trials using supplements, nutraceuticals, and medicinal plants and including studies that evaluate food intake and periodontitis indicators from a multidisciplinary team perspective focused on nutrition, dentistry, and molecular biology. This review aims to raise awareness of the topic and inform the design of new research studies that fill knowledge gaps and support the continued promotion of personalized comprehensive care for these patients. Previous narrative reviews have addressed this topic; however, due to their nature, they have limited methodological rigor in systematically reviewing the literature and generally emphasize nutrient intake regardless of source, not exclusively from food. A limitation of this review is that we did not use the term “periodontitis” on its own; instead, we used the standardized MeSH concept.

The results of this scoping review found three major groups of studies that have established the relationship between nutrient intake and indicators of periodontitis in the adult population: 1) single nutritional components and indicators of periodontitis, 2) groups of nutrients or foods and indicators of periodontitis, and 3) eating patterns and indicators of periodontitis.

In the first case, for a **single nutritional component**, the most relevant finding was that, in a nonsmoking population with chronic periodontitis, protein intake ≥ 1 g/kg/day was associated with lower %BOP values after non-surgical periodontal therapy in a cross-sectional study. Although cross-sectional, the study had strengths, including selecting a population with established periodontitis, classifying them into smokers or nonsmokers, and using quantifiable indicators of periodontitis. In the adjusted model, they considered age, sex, body mass index, and flossing frequency. In addition, the authors used a validated FFQ to approximate dietary intake [30]. In the cohort study (the only one in this section), the principal strength was the 15-year follow-up, which revealed a relationship between fiber intake and a lower risk of alveolar bone loss progression and tooth loss [32].

The other cross-sectional studies found were conducted in the general adult population. They identified those with periodontitis [27,29,31], non-quantifiable periodontitis indicators [29], or a single 24 h recall to assess dietary intake [28,31].

The single nutritional component and periodontitis part included two clinical trial designs, but only Pappe et al. [34] objectively measured the dietary intake, and the Workshop criteria for defining periodontitis and its stages (I–III) were used solely for the general description of the population. In Graziani et al. [33], the diet was measured without a validated instrument. The results were inconsistent after 5 months of twice-daily kiwi fruit intake on periodontal indicators such as the %BOP, CAL, and PI, raising the question of whether the intake of other nutrients differed between groups, not just vitamin C as intended by the authors.

Regarding studies of **nutrient groups or foods** and indicators of periodontitis, the most notable feature was the predominance of assessments of nutrient groups (vitamin A, C, E, K, flavonoid, fiber) alongside plant-based food groups (green–yellow vegetables, general vegetables, fruit). Only a few studies included certain types of fats (DPA, EPA, DHA), carbohydrates, and calories. In almost all of them, they observed negative associations, primarily with CPI, TL, PPD, and CAL. In the only randomized clinical trial evaluating the intake of fruit, vegetables, and whole grains, no clinical differences were found between study groups. However, the authors did not discuss this finding, as it was not the study’s primary objective (Table 2).

This subsection includes two cohort studies [42,43], whose strengths are their follow-up time, oral assessment using quantitative indicators, and diet evaluation through structured instruments and, in one case, validated instruments [43].

We also identified cross-sectional studies and one case–control study [41]. One strength of these studies is their large sample sizes and the use of adjusted multivariate models. However, these methodological designs exhibit biases that should be noted. Some assess dietary intake through single-day recording [35,38] or non-validated questionnaires (or, at least, they did not report validation) [36,37,39] and oral status through the CPI [35,36,37,38] or subjective questionnaires [41]. Therefore, one should interpret the results cautiously.

In this scoping review, the section evaluating **eating patterns** and their relationship with periodontal disease indicators yielded the largest body of evidence, comprising 18 observational studies (Table 3). Adherence to healthy dietary patterns was associated with a lower risk of periodontitis. Conversely, pro-inflammatory dietary patterns significantly heightened the odds of developing periodontal disease.

Prospective cohort studies with substantial sample sizes and follow-up periods of 10 to 24 years provide valuable long-term insights into the protective associations of specific a priori healthy dietary patterns, such as the Elderly Dietary Index (EDI), Healthy Eating Index (HEI), and Empirical Dietary Inflammatory Pattern (EDIP), against clinical parameters of periodontitis.

The main strengths of these prospective cohorts are their large representative samples and extensive follow-up periods, which allow the assessment of temporal sequences and long-term habitual dietary exposure. Furthermore, the systematic use of standardized, validated *a priori* dietary indices, combined with rigorous statistical adjustment for key confounders (e.g., age, smoking, and socioeconomic status), strengthens the validity of these longitudinal associations. Conversely, other longitudinal analyses evaluating *a posteriori* empirical patterns observed no overall protective associations for a Prudent diet or general diet quality. In contrast, a Western dietary pattern was associated with an increased risk of periodontitis, particularly in susceptible subgroups such as obese individuals [60,61]. Additionally, as with many of the aforementioned studies, not all dietary intake data were obtained using previously validated instruments [50,53,57] or standardized indices for determining dietary patterns [53]. Most were designed to monitor health status and, secondarily, adapted to evaluate oral health, limiting the feasibility of obtaining quantitative values for periodontal disease indicators [26,48,54,58].

Researchers have also studied the relationship between eating patterns and clinical parameters of periodontitis in cross-sectional studies. Most studies report beneficial associations between healthy eating patterns and improved periodontal status across various indicators. These studies included a substantial number of subjects, with up to 168,000 participants. The worldwide representativeness of the population (England, Asia, Europe, and America) also stands out. Most studies used previously defined indices, whereas others used statistical techniques to group dietary intake, such as PCA and cluster analysis; some used both, others only PCA, or only clustering. Some studies used instruments to obtain validated dietary data, which enhances data reliability, but most did not, using one instrument or two different instruments [49,50,51,52,55].

Others have linked dietary patterns to the risk of periodontitis; in 2025, Fan et al. [62] conducted a systematic review and meta-analysis including subjects with gingivitis or periodontitis and identified certain patterns. While their meta-analysis pooled 19 studies, our scoping review focused strictly on periodontitis, mapping all identified patterns and overlapping with four of those nineteen studies.

Historically, the lack of standardized definitions of periodontitis has affected epidemiological surveillance and research. Several definitions have been proposed, including the Center for Disease Control and Prevention (CDC) and the American Academy of Periodontology (AAP) from 2007 [63], the definition proposed by Eke in 2012 [64], and the two levels proposed by the European Federation of Periodontology (EFP) [65] (not used in the selected studies); however, none of these proposals had a widespread impact. Finally, the 2017 World Workshop on the Classification of Periodontal and Peri-implant Diseases and Conditions proposed a “case definition system” for periodontitis by both the AAP and the EFP, intended to unify the criteria and reduce the bias and variability in periodontal diagnosis globally [2]. Among the reviewed studies, three [34,39,59] incorporate the periodontitis case definition according to the 2017 Classification and attempt to include elements for staging. Petrenya et al. evaluated the %RBL using orthopantomographic images, despite periapical radiography being better suited to evaluate alveolar bone damage, and defined cases based on %RBL rather than adhering strictly to the case definition based on interproximal CAL, alongside PPD and % BOP [59]. Chuai et al. use the loss of clinical attachment ≥ 5 mm, as their dependent variable, which corresponds to Stage III and IV patients although they did not classify them as such [39]. In turn, Pappe et al. included patients with stage I, II, and III periodontitis in their clinical trial but did not perform an analysis by stage [34].

Alongside diagnostic standardization, controlling for key clinical covariates, particularly oral hygiene, is equally critical in nutritional intake. As periodontal research currently highlights, oral hygiene parameters such as the plaque index, plaque control record, and full-mouth plaque score play a crucial dual role as both confounders and effect modifiers in the relationship between nutrition and periodontal health. Indeed, poor oral hygiene increases the risk of periodontitis by approximately two- to five-fold compared with favorable self-care habits and routine dental checkups [66]. Biofilm control acts as an effect modifier, as the clinical benefits of healthy dietary patterns are significantly more pronounced in individuals with proper oral hygiene.

## 5. Future Perspectives

Epidemiological data are therefore important for establishing a relevant and stable association, but they are insufficient to demonstrate a causal link and, consequently, the general health benefits of treating and preventing periodontitis. Causality can be demonstrated unequivocally only in randomized controlled trials that eliminate or reduce subjects’ exposure to the harmful effects of periodontitis, pathogenic bacteria, and gingival inflammation and control confounding variables related to oral hygiene. These studies must meet the highest quality standards and test therapies that can reduce exposure in a clinically relevant way for each systemic pathology with which a significant association has emerged.

Currently, few studies assess how diet and nutrition can help prevent and/or treat periodontal disease. Therefore, further studies are needed to elucidate the relationship between periodontal disease and diet/nutrition, for example: how long will results last after 4 weeks to 6 months of a healthy diet? What time period of healthy-diet periodontal therapy is needed to achieve better results? Could diet prevent tooth loss?

Future studies on the topic could consider the use of one or more of the following characteristics:

1. Use of visual tools to evaluate the amount of food consumed;
2. Validated tools to determine the intake of specific nutrients;
3. Use of software for nutritional determination that includes representative foods of the region and a wide range of nutrients;
4. Use of artificial intelligence to improve intake estimation;
5. Exploration of eating behaviors and the food environment in conjunction with food intake and variables of lifestyle;
6. Inclusion of other biopsychosocial parameters of nutritional status;
7. Subclassification of macronutrients in statistical analysis of data, for example, carbohydrates (simple, complex, fibrous), proteins (vegetable, animal), and fats (saturated, unsaturated, omega-3 and omega-6 types), groups of antioxidants, fat-soluble vitamins, etc., to clarify their relationship with periodontitis;
8. Classification of periodontal disease using the criteria of the World Workshop carried out in 2017–2018;
9. Wide range of diagnostic measurements (serum parameters, microbiome analysis) for periodontitis;
10. Inclusion of the population of Latin American countries;
11. Longer nutritional interventions (>4 weeks) to know whether significant results in periodontal clinical parameters remain stable.

## 6. Conclusions

In conclusion, this scoping review highlights the link between diet and periodontitis. Among the main findings for a single nutritional component, high protein intake in patients with severe periodontitis was associated with a lower percentage of bleeding on probing; in healthy subjects, intake of good sources of fiber was linked with lower progression of alveolar bone loss. We observed negative associations between nutrient groups (vitamin A, C, E, K, flavonoid, fiber) and plant-based food groups (green–yellow vegetables, general vegetables, fruit) and CPI, TL, PPD, and CAL. Finally, adherence to healthy dietary patterns is associated with mitigated periodontitis risk, and pro-inflammatory dietary patterns were associated with a significantly higher risk of periodontal disease.

Integrating personalized nutritional counseling into routine dental settings represents a promising host-modulating adjunctive therapy to optimize long-term clinical outcomes. Nevertheless, future studies should incorporate the suggestions in this review to advance precision nutrition and dentistry and determine whether personalized dietary strategies serve as preventive or therapeutic measures against periodontitis.

## Figures and Tables

**Figure 1:**
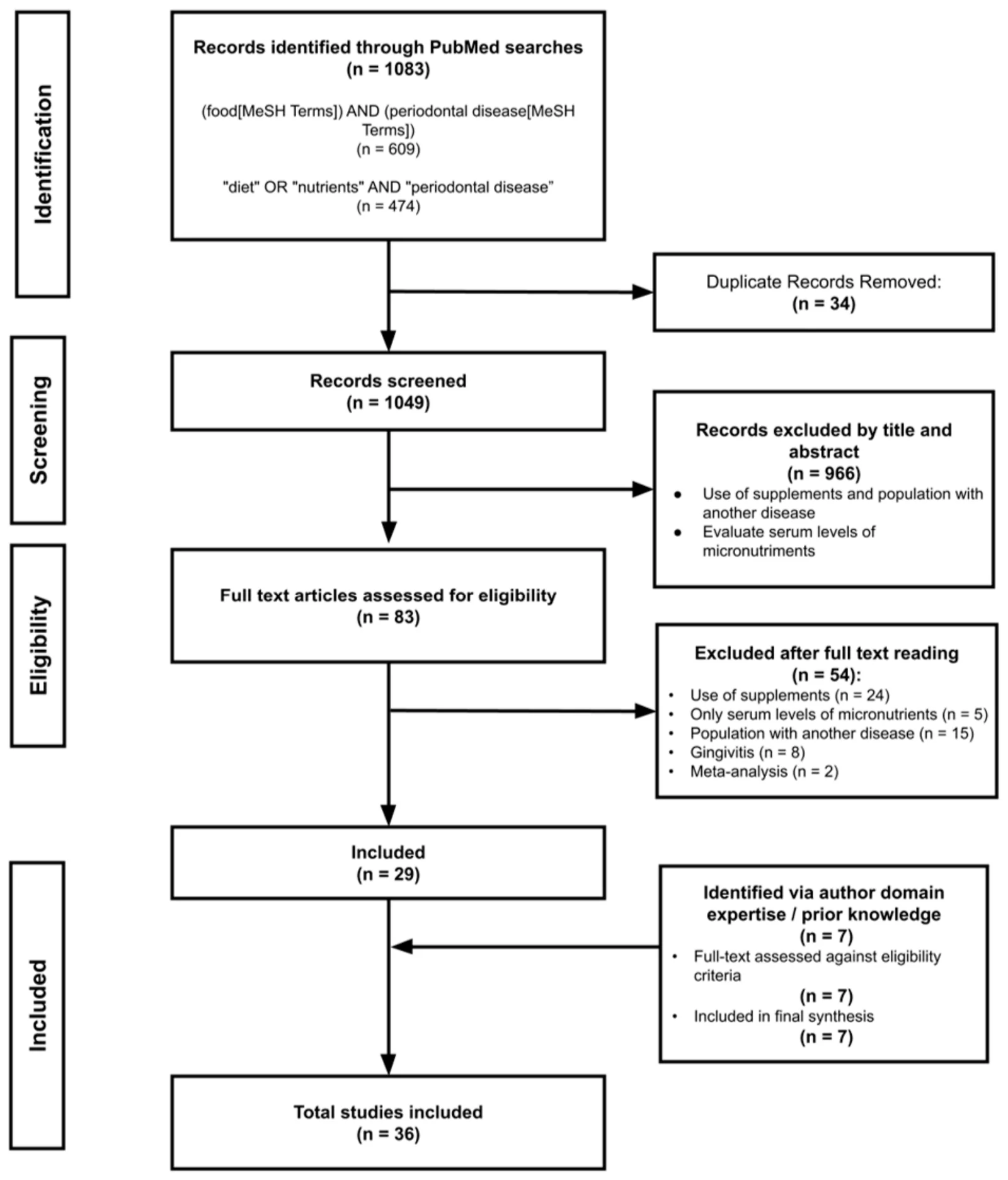
PRISMA flow diagram.

**Figure 2:**
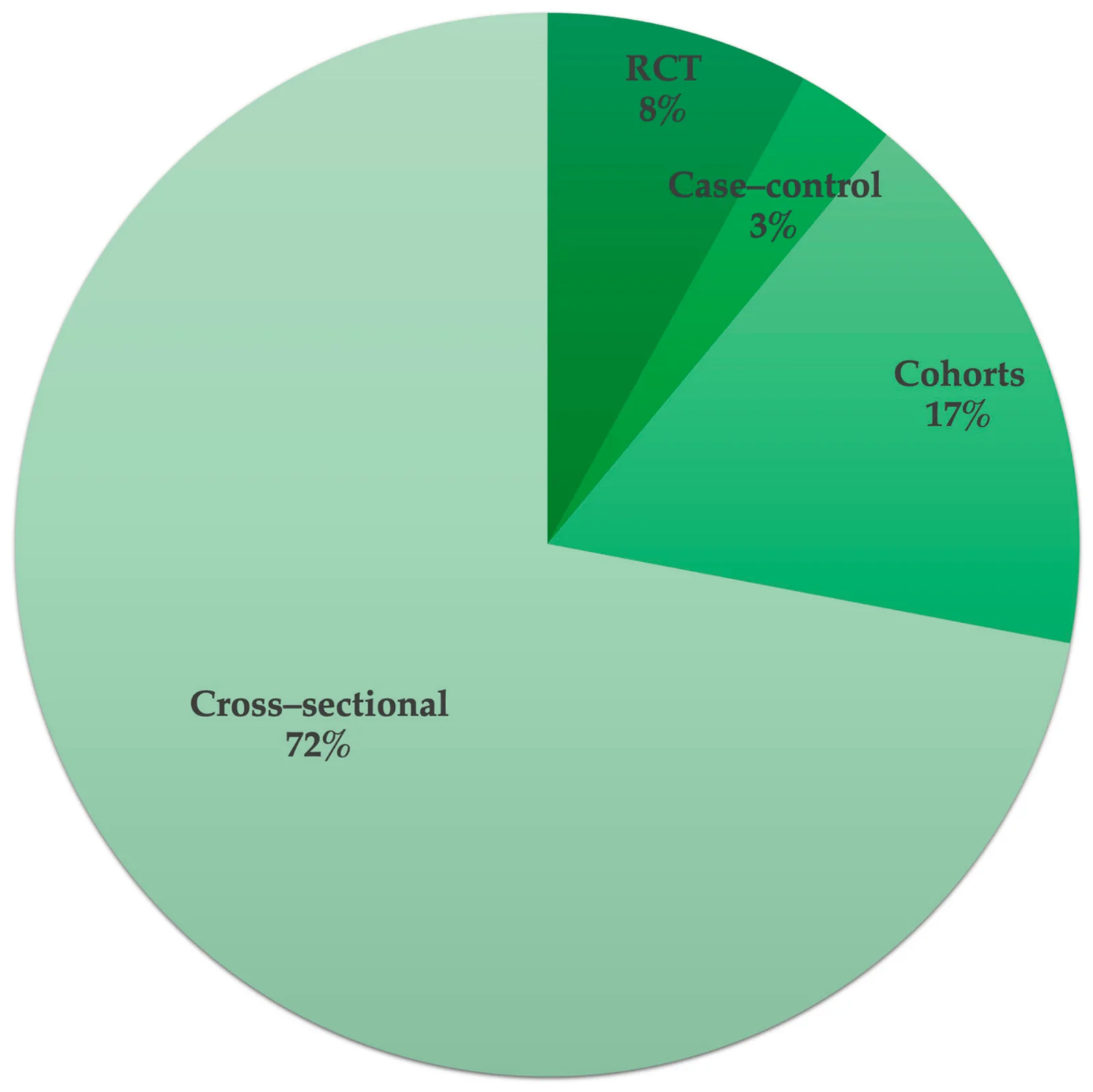
Distribution of included studies by design (percent). RCT = randomized clinical trial.

**Table 1:** Single nutritional component and periodontitis.

First Author, Year	Study Design/Time	Nutrient Evaluated	Sample Size	Population/Condition	Evaluation Tools	Key Findings
1. Alshouibi et al., 2013 [27]	Cross-sectional	Vitamin D	562	Americans/ men, age ≥ 60 y	N: Harvard University FFQ O: PPD, CAL, and ABL	Vitamin D intake was protective against progression of periodontitis. Total vitamin D intake > 800 IU/day was associated with a lower risk of severe periodontitis (OR = 0.67; 95% CI = 0.55–0.81) and moderate-to-severe ABL (OR = 0.54; 95% CI = 0.30–0.96) compared with those who consumed < 400 IU/day.
2. Nielsen et al., 2016 [28]	Cross-sectional	Fiber	6052	Americans/adults, age ≥ 30 y	N: 24 h recall O: PPD and CAL	After adjusting for confounding variables, the lowest quartile (≤11.9 g fiber/d) was associated with moderate–severe periodontitis vs. the highest quartile (>23.25 g fiber/d) (OR = 1.30; 95% CI: 1.00–1.69). Consuming whole grains in the lowest quartile (0-ounce equivalents) was associated with severe periodontal disease (OR = 1.32; 95% CI:1.08–1.62). Fruit and vegetable intake was not associated with periodontitis severity.
3. Lee et al., 2017 [29]	Cross-sectional	Dietary vitamin C	10,930	Koreans/ adults, age ≥ 19 y	N: 24 h recall O: CPI	Inadequate dietary intake of vitamin C (<75 mg/day) increased the risk of periodontitis (OR = 1.161; 95% CI: 1.042–1.294).
4. Dodington et al. 2021 [30]	Cross-sectional	Protein	Nonsmokers = 63 Smokers = 23	Canadians/ adults, age ≥ 50 y with chronic periodontitis	N: Validated FFQ O: PPD, BOP and PI	Nonsmokers who ate ≥ 1 g/kg/day of protein and used dental floss more often presented fewer BOP sites than those who ate <1 g/kg/day (BOP sites: 3 ± 6 vs. 8 ± 9; *p* = 0.009). After adjusting for confounding variables, those who ate ≥ 1 g/kg/day of protein had fewer sites with probing depth ≥ 4 mm after SRP (8 ± 2 vs. 18 ± 2; *p* < 0.01; β coefficient = −9.7; 95% CI: −15.5, −3.9). No associations or differences were seen in smokers.
5. Guo et al., 2025 [31]	Cross-sectional	Whole grains	7753	Americans/adults, NHANES age ≥ 30 y	N: 24 h recall O: CAL and PPD	The whole grains intake (Q4 OR: 0.70 95%; CI: (0.56–0.89)) and whole/refined grain ratio (Q4 OR: 0.75 (95%; CI: 0.60–0.93)) were negatively associated with periodontitis.
6. Schwartz et al., 2012 [32]	Cohort 15-year follow-up	Fiber	625	Americans/ men, age ≥ 53–74 y	N: Harvard University FFQ O: number of teeth remaining, levels of calculus, BOP, PPD and ABL	Good to excellent sources of fiber (2.5 g per serving), especially fruits, were associated with a lower risk of ABL progression (HR = 0.76; 95% CI: 0.60–0.95) and tooth loss (HR = 0.72, 95% CI: 0.53–0.97).
7. Graziani et al., 2018 [33]	Randomized controlled trials 5 months	Kiwifruit	Control = 25 Experimental = 25	Italians age ≥ 18 y with periodontitis and self-reported as systemically healthy	N: Self-report of the daily intake of kiwifruit O: PPD, CAL, BOP, and PI	The consumption twice/daily of kiwifruit reduces gingival inflammation (before SRP) at 2 M and BOP (6.67% ± 11.90, *p* = 0.01). At 2 M, the experimental group after adjusted showed lower values of BOP (36.16 ± 20.39 vs. 51.49 ± 15.99; *p* = 0.009), PI (51.40 ± 20.92 vs. 65.10 ± 13.97; *p* = 0.014) and CAL (0.66 ± 0.46 vs. 0.89 ± 0.83; *p* = 0.031). After 5 M (after SRP), the control group presented a larger reduction in BOP, PI, and CAL than the test group. Therefore, kiwi consumption did not provide additional benefits beyond periodontal therapy.
8. Pappe et al., 2025 [34]	Randomized controlled trials 4 weekw	Sugar	Control = 11 Sugar avoidance group = 10	Germans/ age 18–75 y, generalized periodontitis	N: FFQ O: GBI, PPD, PCR and BOP	After 4 weeks of sugar avoidance, BOP decreased significantly in the no-sugar group (40.3% ± 15.54) and by 34% ± 12.47 in the control group (intra-group *p*-value < 0.001 in both cases). PCR and GBI decreased significantly in both groups. In none of the cases, differences were observed between the groups.

ABL; alveolar bone loss, BOP; bleeding on probing, CAL; clinical attachment loss, CPI; Community Periodontal Index, FFQ; food frequency questionnaire, GBI; Gingival bleeding index; PCR; plaque control record, PI; plaque index, PPD; periodontal pocket depth, SRP; scaling and root planing, N; nutritional, O; oral, y; years.

**Table 2:** Group of nutrients or foods and periodontitis.

First Author, Year	Study Design/Time	Group of Nutrients or Foods Evaluated	Sample Size	Population/Condition	Evaluation Tools	Key Findings
1. Hamasaki et al., 2017 [35].	Cross-sectional	Macronutrients and micronutrients	3043	Japanese/ adults, age ≥ 20 y	N: 24 h recall O: CPI	Adjusted multivariate logistic regression with CPI as the response variable showed that fat intake is associated with periodontal disease severity (β coefficient = 0.984; *p* = 0.026). The percentage of fat intake was higher in the CPI 0–2 group vs. CPI 3–4 (25.2 vs. 23.2; *p* < 0.001).
2. Hosoda et al., 2021 [36]	Cross-sectional	Food and beverage habitual consumed	120	Japanese/women, age ≥ 20.4 y healthy college nonsmokers	N: Diet history questionnaire (includes FFQ, semi-quantitative and general eating habits) O: CPI	The multivariate logistic regression analysis in young adult women evaluated for periodontitis, using the CPI as response variable showed an inverse relation with vitamin E (OR = 0.528; 95% CI; 0.284–0.985) and green and yellow vegetables (OR = 0.982; 95% CI; 0.966–0.997) intake with periodontitis.
3. Kwon and Kim, 2022 [37]	Cross-sectional	Plant foods	4514	Koreans/ elderly people, age ≥ 65 y	N: Dietary behaviors from the food insecurity (dietary survey questionnaire) and 24 h recall O: CPI	In the adjusted model for confounding variables they found a relation between fruit intake and periodontitis (OR = 0.733; 95% CI: 0.579–0.929). Intake was higher in the non-periodontitis group than in the periodontitis group (138 vs. 106 g/day; *p* = 0.0013).
4. Hwang et al., 2022 [38]	Cross-sectional	Phosphorus, riboflavin, thiamine, niacin, vitamin C, calcium, protein, carbohydrates, and fat	12,689	Koreans/ adults, age ≥ 19 y	N: 24 h recall O: CPI	Adjusted multivariate logistic regression shows an inverse association between phosphorus (OR = 0.88; 95% CI: 0.66–0.97), carbohydrate (OR = 0.85; 95% CI: 0.70–0.98), and fat (OR = 1.41; 95% CI: 1.13–1.75) intake and periodontitis.
5. Chuai et al., 2023 [39]	Cross-sectional	Vitamin K and fiber	4965	Americans/NHANES from 2009 to 2014 adults, age ≥ 30 y	N: instrument was not reported O: CAL and PPD	Vitamin K and fiber intake were negatively associated with CAL progression, (β = −0.248; 95% CI: −0.33; −0.16; β = −0.28; 95% CI: −0.36; −0.20 respectively). After adjusting for confounders, these associations remained stable.
6. Zhang et al., 2025 [40]	Cross-sectional	Vitamins: A, B1, B2, B6, B12, C, D, E, and K	7708	Americans/ healthy adults NHANES III (2009–2014) age, ≥ 30 y	N: Two 24 h recalls. O: CAL, PPD	Vitamin A (OR = 0.90; 95% CI: 0.95–0.99) and vitamin E intake (OR = 0.95; 95% CI: 0.93–0.97) were negatively associated with periodontitis. Excessive intake (>75th percentile) of the vitamin mixture did not significantly reduce the odds of periodontitis.
7. Mewes et al., 2022 [41]	Case–control	Vitamins and fatty acids	Controls = 326 Cases = 326	Germans/ age ≥ 50 y with and without periodontitis	N: Nutrition FFQ by German Institute for Human Nutrition in Potsdam O: Questionnaires (yes/no answers)	Periodontal patients showed a significantly higher intake of DPA (*p* = 0.003) and vitamin C (median difference: 9.24%, *p* = 0.02) regardless of tooth loss (TL) (TL, and specifically in DPA without TL and vitamin C in patients with TL). DPA serum concentration was higher in periodontitis than in controls (OR = 1.52; 95% CI: 1.15–2.01). EPA was higher in serum levels of periodontitis compared to controls (OR = 1.28; 95% CI: 1.04 –1.58).
8. Dodington et al., 2015 [42]	Cohort 1-year follow-up	Fruits, vegetables, antioxidants and anti-inflammatory nutrients	Nonsmokers = 63 Smokers = 23	Canadians/adults age ≥ 50 y with chronic periodontitis,	N: FFQ and dietary supplement use questionnaire O: PPD, PI, and BOP	Nonsmokers with higher intake of fruits and vegetables, β-carotene, vitamin C, α-tocopherol, EPA, and DHA had a lower percentage of areas with PPD > 3 mm after SRP. Reverse associations were observed between PD (% sites >3 mm) and dietary b-carotene (β coefficient = −0.29; 95% CI: −0.53, −0.06), vitamin C (β = −0.36; 95% CI: −0.58, −0.14) and total EPA + DHA (β coefficient = −0.28; 95% CI: −0.52, −0.04).
9. Sparrow et al., 2020 [43]	Cohort 3–4 years following SRP	Vitamin C and flavonoids	34	Canadians/age ≥ 50 y, moderate to severe periodontal disease	N: Validated FFQ O: PPD, BOP, PI and tooth mobility	Flavonoid intake (foods and beverages) was negatively associated with PPD (r = −0.36; *p* = 0.03) post-SRP among patients that receive regular periodontal maintenance.
10. Zare Javid et al., 2014 [44]	Randomized controlled trial 3 and 6 months after the intervention	Fruit, vegetables and whole grains	Control = 19 Intervention = 18	British/adults age ≥ 30–65 y with chronic periodontitis,	N: 3-day estimated food diary O: PI, PPD, GR, BI, and CAL	Higher intakes of fruit and vegetables showed no significant differences in periodontal indices (PI, PPD, GR).

BI; bleeding index, BOP; bleeding on probing, CAL; clinical attachment loss, CPI; Community Periodontal Index, DPA; docosapentaenoic acid, DHA; docosahexaenoic acid, EPA; eicosapentaenoic acid, FFQ; food frequency questionnaire, GR; gingival recession, N; nutritional, NHANES; National Health and Nutrition Examination Surveys, O; oral, PI; plaque index, PPD; periodontal probing depth, SRP; scaling and root planing, TL; tooth loss, y; years.

**Table 3:** Eating patterns and periodontitis.

First Author, Year	Study Design/Time	Eating Patterns Evaluated	Sample Size	Population/Condition	Evaluation Tools	Key Findings
1. Jauhiainen et al., 2016 [45]	Cross-sectional	Nordic Diet	2187	Finns/adults age 30–49 y, 50–79 y without risk factors (smoking, diabetes, rheumatoid arthritis),	N: FFQ and two a priori indices to determine dietary patterns BSDS and RFDS O: A field-dentist evaluated oral health and took parallel measurements of PPD, BOP, level of dental plaque (Silness and Löe)	Higher RFDS (PRR = 0.98; 95% CI: 0.96–0.99) scores were associated with fewer sextants with BOP in younger adults with poor oral hygiene. In the older age group with poor oral hygiene, both BSDS and RFDS associated inversely with the number of sextants with gingival bleeding [(PRR = 0.98; 95% CI: 0.97–0.99) and (PRR = 0.97; 95% CI: 0.96–0.99) respectively] and with the number of teeth with PPD [(PRR = 0.96; 95% CI: 0.94–0.98) and (PRR = 0.96; 95% CI:0.94–0.99) respectively].
2. Jauhiainen et al., 2018 [46]	Cross-sectional	Nordic Diet	971	Finns/Adults age 30–49 y, 50–79 y without risk factors (diabetes and rheumatoid arthritis), daily smokers	N: FFQ and two a priori indices to determine dietary patterns BSDS and RFDS O: A field-dentist evaluated oral health and took parallel measurements of PPD and BOP, level of dental plaque (Silness and Löe)	In the adjusted models, scores were not associated with the number of sextants with BOP or teeth with PPD in any age group. In the younger age group, teeth with PPD were weakly inversely associated with RFDS (PRR = 0.63; 95% CI: 0.43–0.92) among those with good oral hygiene. No associations were observed between dietary indices and periodontal parameters in the older age group with good oral hygiene.
3. Salazar et al., 2018 [47]	Cross-sectional	Diet quality	13,920	Hispanics/population in the United States, age ≥ 18–74 y	N: Two 24 h recalls and AHEI-2010 for dietary pattern O: 18 dental examiners performed full- mouth periodontal examinations CAL, PD and BOP	A higher AHEI score (better diet quality) was associated with a lower risk of severe periodontitis (CAL) in an adjusted model (OR highest versus [vs.] lowest quartile score = 0.40; 95% CI: 0.28–0.58), with evidence of a significant dose–response relationship (*p* < 0.0001).
4. Jauhiainen et al., 2020 [48]	Cross-sectional	Nordic Diet	240	Finns/adults age 30–49 y, 50–79 y without risk factors (smoking, diabetes, rheumatoid arthritis)	N: FFQ and two a priori indices to determine dietary patterns BSDS and RFDS O: A field-dentist evaluated oral health and took parallel measurements of PPD, level of dental plaque (Silness and Löe)	Low scores (indicating poor diet) in both the BSDS and the RFDS were associated with a higher number of teeth with ≥4 mm PPD (BSDS: IRR = 0.94; 95% CI: 0.92–0.96, RFDS: IRR = 0.91; 95% CI: 0.88–0.94). This association was stronger in nonsmokers than in the whole study (BSDS: IRR = 0.85; 95% CI: 0.82–0.88; RFDS: IRR = 0.74; 95% CI: 0.71–0.77).
5. Wright et al., 2020 [49]	Cross-sectional	Real-world dietary patterns	10,010	Americans/NHANES, age ≥ 30 y	N: Two 24 h recalls, treelet transformation of a hybrid of hierarchical clustering and PCA for dietary patterns O: Dental examiners were trained and calibrated by the survey’s reference examiner. They measured GR, PPD, and CAL	In the population study, eight dietary patterns were observed. A high adherence to dietary pattern rich in salads, fruits and vegetables, poultry, seafood, and water or tea to drink was associated with a lower CAL extent (8.1% or < 3 mm) compared with low adherence (OR, decile 10 compared with decile 1 = 0.67; 95% CI: 0.46–0.99) in the adjusted model. The other seven patterns were not associated with CAL extent.
6. Costa et al., 2021 [50]	Cross-sectional	Micronutrient patterns	450	Brazilians/adults, age ≥ 18 y	N: FFQ, PCA and cluster analysis O: PSR	The group of “Poor Periodontal Status” was represented by lower intake of fiber, potassium, copper, magnesium, manganese and vitamin C (*p* <0.001). In comparison, the “Healthy Periodontal Status” group was characterized by a higher intake of energy, zinc, fiber, iron, magnesium, manganese, phosphorus, potassium and omega 3 (*p* < 0.001), which was associated with better periodontal health (PSR < 3).
7. Li et al., 2021 [51]	Cross-sectional	Inflammatory diet	7081	Americans/NHANES, age ≥ 30 y	N: Two 24 h recalls and E-DII O: Dental examiners were trained and calibrated by the survey’s reference examiner. They measured GR, PPD, and CAL	A higher E-DII score was associated with a higher risk of periodontitis (using PPD and CAL) ([OR tertile3 vs. 1] = 1.53; 95% CI: 1.33–1.77). Models were adjusted for age, gender, race/ethnicity, education, income, smoking habit, alcohol consumption, physical activity, dental visit, abdominal adiposity, obesity, hypertension, dyslipidemia, dysglycemia, WBC count, cardiovascular disease, arthritis, total energy intake, and the number of teeth present.
8. Machado et al., 2021 [52]	Cross-sectional	Inflammatory diet	10,178	Americans/NHANES, age ≥ 18 y	N: Dietary interview and 24 h recall period before the interview and DII O: Dental examiners were trained and calibrated by the survey’s reference examiner. They measured GR, PPD, and CAL	Positive significant correlation was observed among DII scores and with mean PPD, CAL, and thresholds of PPD and CAL (*p* < 0.001). A linear regression adjusted for multiple confounding variables confirmed the association between DII scores and mean PPD (β coefficient = −0.06, SE = 0.01, *p* < 0.001), mean CAL (β coefficient = −0.08, SE = 0.01, *p* < 0.001), n sites PPD ≥ 5 (β coefficient = −0.01, SE = 0.00, *p* < 0.001), and n sites CAL ≥ 5 (β coefficient = −0.02, SE = 0.00, *p* < 0.001).
9. Altun et al., 2021 [53]	Cross-sectional	DASH and Mediterranean patterns	6209	Germans/Hamburg City age ≥ 50 y Health Study with none-mild, moderate and severe periodontitis	N: FFQ validated DASH adherence score and MEDAS O: Full dental examination by nurses trained regularly by a dentist. MPD, GR, BOP, PI, DMFT and CAL	Consumption of the DASH diet (OR = 0.95; 95% CI: 0.89–1.00; *p* < 0.0507) and the MEDAS (OR = 0.97; 95% CI: 0.94–1.00) was associated with a lower risk of periodontitis in adjusted models. Poor adherence to the DASH diet and the MEDAS showed higher values of BOP, PI, and DMFT compared with high adherence (*p* < 0.001 for all).
10. DeMayo et al., 2021 [54]	Cross-sectional	Diet quality	923	Americans/healthy adults, age ≥ 20–55 y, Glucose Intolerance and Insulin Resistance Study (ORIGINS)	N: FFQ validated AHEI and APDQS for diet quality O: Trained examiner measured PD, MAL, BOP, and MPD	AHEI and APDQS were not associated with MPD, MAL, or periodontitis. Increased adherence to APDQS was associated with decreased BOP after multivariable adjustment (*p* < 0.03)
11. Watson et al., 2022 [55]	Cross-sectional	Nutrient-based dietary patterns (high micronutrient and fiber, high fat, high protein, and high sugar)	9476	British/adults, age ≥ 40–69 y	N: FFQ and 24 h recall and PCA for dietary patterns O: Self-reported periodontal disease	Among the four eating patterns were evaluated, the high micronutrient and fiber pattern was inversely associated with periodontal disease risk (defined by symptoms of painful gums, bleeding gums, or loose teeth), remaining significant after multiple adjustment (OR = 0.76; 95% CI: 0.65–0.90). Conversely, higher intakes (Q3 and Q4 vs. Q1) of the high fat pattern were initially associated with increased risk (OR = 1.17; 95% CI: 1.00–1.36), but this association did not remain after adjustment (OR = 1.13; 95% CI: 0.97–1.32). No associations were observed for the high-sugar or high-protein patterns.
12. Choi et al., 2023 [56]	Cross-sectional	Inflammatory diet	168,378	Koreans/KoGES healthy adults age ≥ 40–69 y	N: SQ-FFQ validated, DII, E-DII scores O: Diagnosis (not by a dental specialist)	Cox proportional hazard models revealed a significantly increased risk of incident periodontitis in individuals consuming high E-DII diets in the total population (HR quartile 4 vs. 1 = 1.29; 95% CI: 1.13–1.48) and in both men (HR quartile 4 vs. 1 = 1.36; 95% CI: 1.07–1.73) and women (HR quartile 4 vs. 1 = 1.27; 95% CI: 1.08–1.50).
13. Li et al., 2023 [57]	Cross-sectional	Plant-based diet	5651	Americans/healthy adults NHANES III age ≥ 40 y	N: FFQ, hPDI, uPDI O: Partial mouth periodontal examination protocol PPD, CAL, BOP, and NMT	They observed an inverse association of hPDI z-score and periodontitis (OR = 0.925; 95% CI: 0.860–0.995). Higher uPDI z-score (adherence to unhealthful plant foods) might increase the risk of periodontitis (OR = 1.100; 95% CI: 1.043–1.161). Regarding clinical parameters, they observed an inverse association between hPDI z-score and mean PPD, CAL, and BOP. In contrast, uPDI z-score was positively associated with these.
14. Lieske et al., 2023 [58]	Cross-sectional	Anti-inflammatory diet	5642	Germans/healthy adults age 45–75 y	N: FFQ validated, and anti-inflammatory dietary score (developed by the authors) O: PI, BOP, PPD, CAL and DMFT Index Calibrated examiners, under dentist supervision, measured these clinical variables	A significant association between a higher anti-inflammatory dietary score and lower odds to present periodontitis in an adjusted model (age, sex, smoking, diabetes, hypertension, and physical activity) (OR = 0.93; 95% CI: 0.89–0.98).
15. Petrenya et al., 2024 [59]	Cross-sectional	Multiple dietary patterns	1487	Norwegians/healthy adults, age ≥ 40–79 y	N: SQ-FFQ (Oslo University validated), and PCA form pattern definition O: PPD, %RBL by calibrated dental hygienists orthopantomogram	The authors found four eating patterns; “fruit and vegetables”, “westernized”, “meat/fish and potatoes”, and “refined grains and desserts”. The fruit and vegetables pattern was inversely associated with periodontitis (rapid bone loss vs. slow bone loss; OR = 0.49; 95% CI: 0.25–0.98). The group with a higher consumption of refined grains and desserts had a higher risk of having fewer teeth (OR = 3.52; 95% CI: 1.69–7.33).
16. Alhassani et al., 2021 [60]	Cohort 24 years of follow-up	Prudent and Western patterns	34,940	Americans/health professional study, male age ≥ 40–75 y	N: FFQ and PCA for dietary patterns O: Self-reported periodontal disease	No overall relation was observed between Prudent or Western dietary patterns and periodontal disease risk. Although the highest quintile of the Western pattern initially showed a weak association in the age-adjusted model (HR = 1.18; 95% CI: 1.07–1.30), this association became non-significant in multivariate adjusted models. However, among obese individuals, those in the highest Western pattern quintile presented an 83% higher risk of periodontal disease compared to the lowest quintile in the adjusted model (HR = 1.83; 95% CI: 1.21–2.76).
17. Alhassani et al., 2021 [61]	Cohort 24 years of follow-up	Empirical dietary inflammatory pattern	34,940	Americans/male health professionals age ≥ 40–75 y	N: FFQ and an EDIP using RRR O: Self-reported periodontal disease	EDIP scores showed no overall association with the risk of periodontal disease in the analysis; the HR in the highest quintile of EDIP was 1.01 compared to the lowest quintile (95% CI: 0.90–1.12). Obese men nonsmokers in the highest quintile of EDIP had 39% more risk of periodontitis than those in the lowest quintile of EDIP (HR = 1.39; 95% CI: 0.98–1.96)
18. Kotronia et al., 2021 [26]	Cohort 10 years of follow-up	Diet quality	BRHS: 2147 HABC: 2998	BRHS/males, 71–92 y and HABC, British and Americans females and males, 71–80 y	N: BRHS (FFQ validated and EDI) and HABC (FFQ and HEI) O: Objective markers (oral examination) included count of natural teeth and periodontal disease measures (CAL and PPD). Subjective (self-rated) oral health markers (questionnaires)	In the BRHS population, having 8–14 teeth (OR = 1.57; 95% CI: 1.05–2.34), fair/poor self-rated oral health (OR = 1.51; 95% CI: 1.14–1.99), and an accumulation of oral health problems (OR = 1.83; 95% CI: 1.11–3.01) were associated with poor diet quality after adjusting for confounders. In the age-adjusted model over a 10-year follow-up (age 60–79 to 71–92 years), a persistent low EDI score was associated with partial tooth loss (<21 teeth vs. ≥21), periodontal disease, fair/poor self-rated oral health, and accumulation of oral health problems. In the HABC population, tooth loss and periodontal disease (CAL) were associated with poor diet quality in age-adjusted models, but these associations did not remain after complete adjustment.

AHEI; Alternative Healthy Eating Index, APDQS; A Priori Diet Quality Score, BOP; bleeding on probing, BRHS; British Regional Heart Study, BSDS; Baltic Sea Diet Score, CAL; clinical attachment lost, CI; confidence interval, DASH; Dietary Approach to Stop Hypertension, DII; Dietary Inflammatory Index, DMFT; decayed missing filled teeth, EDI; Elderly Dietary Index, E-DII; Energy-adjusted Dietary Inflammatory Index, EDIP; empirical dietary inflammatory pattern, FFQ; Food Frequency Questionnaires,, GR; gingival recession, HABC; Health Aging and Body Composition Study, HEI; Healthy Eating Index, hPDI; Healthy Plant-Based Diet Index, HR; hazard ratio, IRR; incidence rate ratios, KoGES; Korean Genome and Epidemiology Study, MAL; mean attachment loss, MEDAS; Mediterranean adherence score, MPD; mean probing depth, NMT; number of missing teeth, NHANES; National Health and Nutrition Examination Survey, PCA; principal component analysis, PI; Plaque Index, PD; probing depth, PPD; periodontal pocket depth, PRR; prevalence rate ratios, PSR; periodontal screening and recording, RRR; reduced rank regression, %RBL; per cent radiographic marginal bone level, RFDS; Recommended Finnish Diet Score, SQ-FFQ; Semiquantitative Food Frequency Questionnaire, uPDI; Unhealthy Plant-Based Diet Index, WBC; white blood cell, N; nutritional, O; oral, OR; odds ratio.

## Data Availability

No new data were created or analyzed in this study. Data sharing does not apply to this article.

## Supplementary Materials

The following supporting information can be downloaded at https://www.mdpi.com/article/10.3390/nu18213537/s1, Table S1: Preferred Reporting Items for Systematic Reviews and Meta-Analyses extension for Scoping Reviews (PRISMA-ScR) Checklist.
